# Supplementary figures and images for: Characteristic Immune Dynamics in COVID-19 Patients with Cardiac Dysfunction
Source: J Clin Med. 2022 Mar 28;11(7):1880. doi: 10.3390/jcm11071880 (PMC8999785; doi:10.3390/jcm11071880)

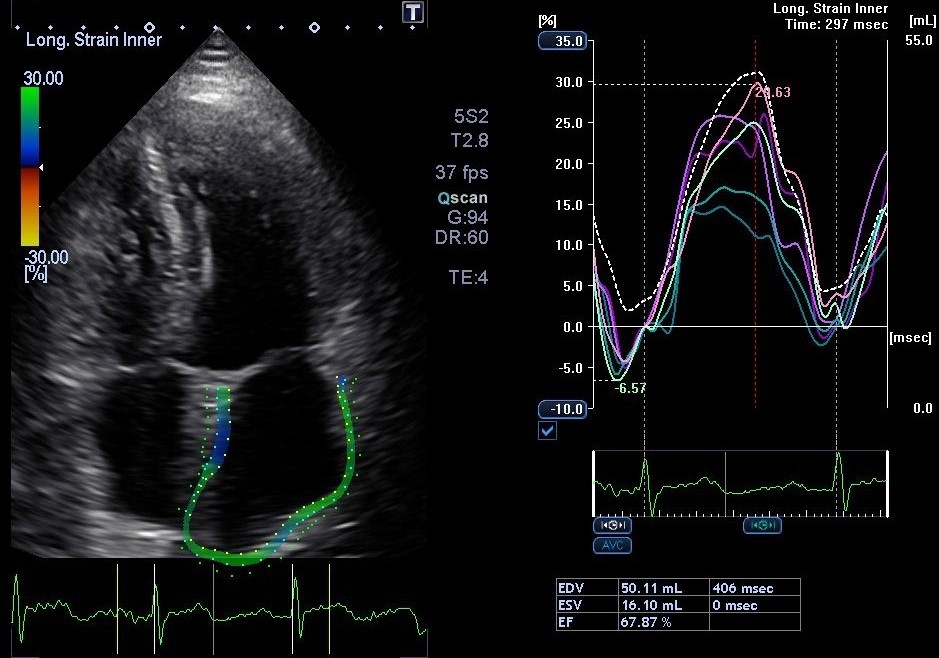

Supplement: Supplementary file 1 [file jcm-11-01880-s001.zip › Figure S1.jpg]

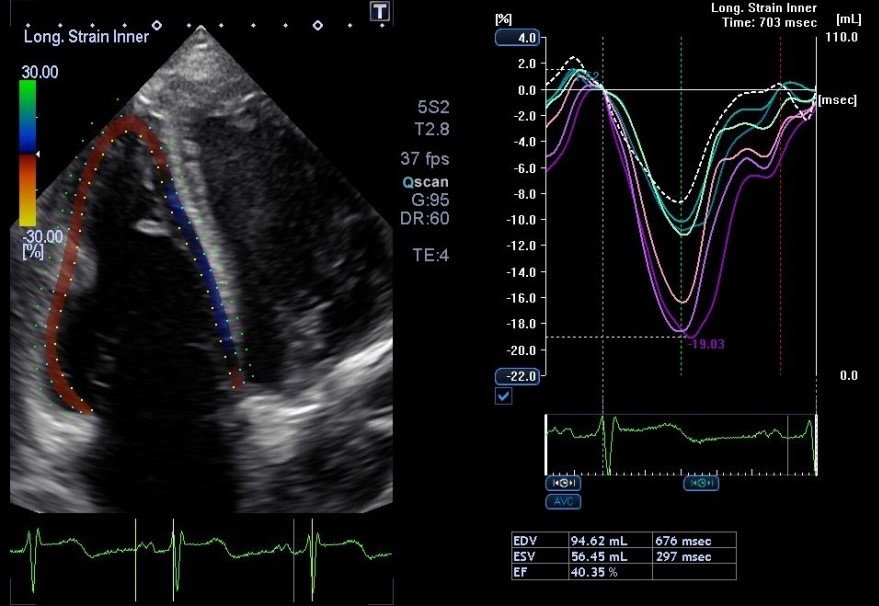

Supplement: Supplementary file 1 [file jcm-11-01880-s001.zip › Figure S2.jpg]

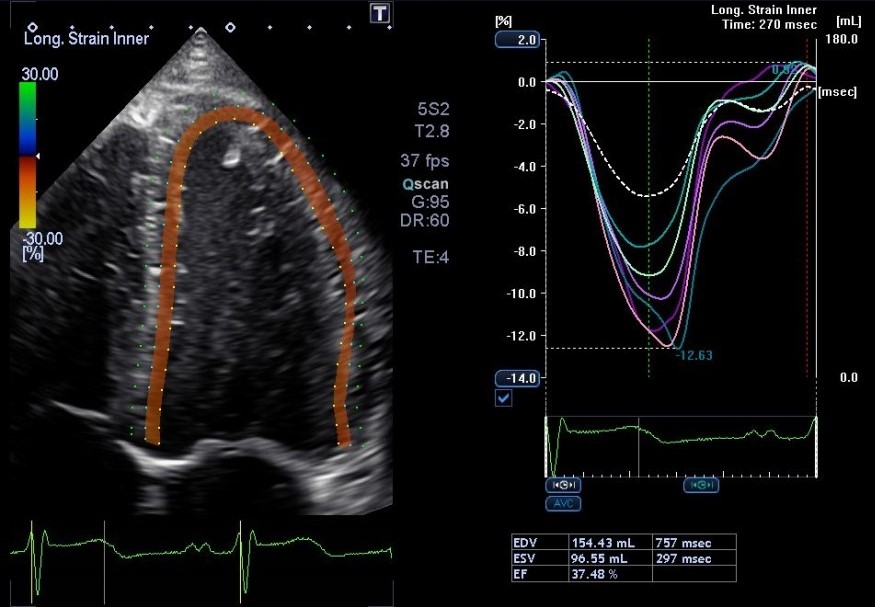

Supplement: Supplementary file 1 [file jcm-11-01880-s001.zip › Figure S3.jpg]
